# Central Giant Cell Granuloma: A potential endodontic misdiagnosis

**Published:** 2009-10-10

**Authors:** Safoura Seifi, Ramin Fouroghi

**Affiliations:** 1 *Department of Oral and Maxillofacial Surgery, Babol University of Medical Sciences, Babol, Iran.*

**Keywords:** Central Giant Cell Granuloma, Dental Granuloma, Periapical disease, Radicular cyst

## Abstract

Central Giant Cell Granulomas (CGCGs) may manifest as radiolucencies anywhere in the mandible or maxilla. In rare cases, it can appear as a localized periradicular area and mimic an endodontic lesion. This case report presents an uncommon location of CGCG which was not accurately diagnosed nor timely treated. Periodic follow ups of periapical radiolucencies after RCT are necessary. Dentists should include CGCG in differential diagnosis of lesions that are refractory to endodontic treatment. [Iranian Endodontic Journal 2009;4(4):158-60]

## INTRODUCTION

Central Giant Cell Granuloma (CGCG) is an uncommon benign lesion (1) that was described by Jaffe in 1993 (2). There are some controversies about its nature; some associate it as benign tumor of the maxillofacial skeleton (3), others as reactive, non neoplastic lesion (4). CGCGs are more commonly found in the mandible and mainly in young adults (1). Clinical and radiographic appearances of CGCG are not pathognomonic (5). Two distinct forms are currently recognized; 1) nonaggressive and asymptomatic slow growing forms which do not perforate cortical bone and 2) aggressive forms that result in expansion and perforation of cortical bone and even tooth displacement and resorption (6). CGCGs have various radiographic appearances; most lesions are multilocular, well circumscribed, and noncorticated radiolucencies. However, they may occasionally manifest as unilocular corticated radiolucencies (5). The anterior segment of mandible is affected more commonly than other regions (1).

Aggressive and non-aggressive forms of CGCGs are similar in their histopathologic features (6) which demonstrate lobules of spindle fibroblasts, numerous multinucleated osteoclast-like giant cells and hemorrhage and reactive woven bone rimmed by osteoblasts. In addition, scattered inflammatory cells within the stroma can be seen (5,6).

CGCGs that are localized to the periapical region or lateral to tooth roots can be easily confused with inflammatory odontognic lesions such as dental granuloma and radicular cyst. The common occurrence of periapical granulomas and cysts lead the clinician to arrive at a definitive diagnosis without full diagnostic tests and histopathologic examination (7). This case report discusses a CGCG that appeared as periapical radiolucency associated with a mandibular right canine which was initially misdiagnosed and treated as a radicular cyst.

## CASE REPORT

**Figure 1:**
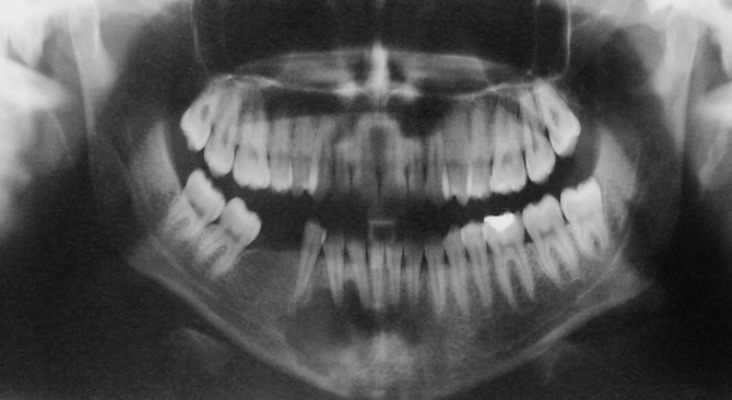
In panoramic radiography was revealed unilocular radiolucency in periapical area of anterior mandibular teeth.

A 30 year-old woman was seen in a dental surgery complaining of painful swelling on her anterior lower jaw in the parasymphysis area. The history of complaint revealed gradual growth of the swelling during the past year. The medical history was not significant. Oral cavity examination revealed a 2.5-cm fixed painful mass in anterior of mandible. The oral mucosa was intact. Intraorally, anterior lower teeth were intact and not mobile. Panoramic and periapical radiographies revealed well demarcated unilocular periapical radiolucency in region of mandibular incisors and canine (Figure 1). 

**Figure 2:**
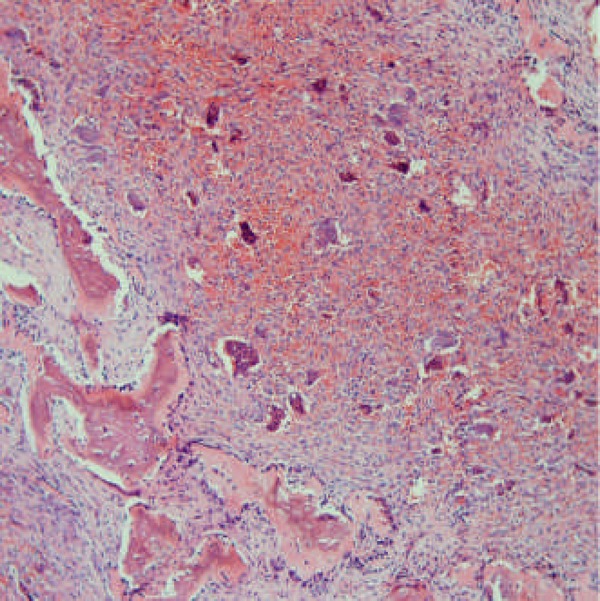
Histopathologic view with H&E staining in CGCG (×10)

Sensitivity tests of involved teeth were negative for the right mandibular canine.Initially, root canal treatment of right mandibular canine was performed. However, after 6 months, expansion and pain of anterior region of mandible had increased gradually.

The patient was referred to an endodontist for re-treatment of right mandibular canine. After re-treatment, signs and symptoms did not subside and the swelling of the anterior mandible persisted. Patient was referred to oral and maxillofacial surgeon. Needle aspiration biopsy was carried out, unfortunately it demonstrated negative results.

Clinical examination discovered no neurosensory defects by the oral surgeon. These findings suggested a differential diagnosis of Central Giant Cell Granuloma (CGCG), Aneurysmal Bone Cyst, or Cystic Calcifying Odontogenic Tumor. Excisional biopsy was conducted.

Gross examination of lesion revealed many small soft elastic specimens, white-brown in color with size of 2.5 2 0.5 cm. Solid section surface of specimens was seen.

Histologically, multinucleated giant cells were distributed in a stroma that was highly cellular compromising both spindle-shaped and round cells which were found mostly in hemorrhagic and reactive bone areas. Ingested RBCs and scant collagen fibers were also seen (Figure 2). These findings confirmed diagnosis of CGCG. After excisional biopsy and curettage, 7-months follow up did not show recurrence.

## DISCUSSION

CGCG is a benign intra-osseous lesion with unknown etiology (8). A quantity of studies suggests local trauma, sex hormones and genetics as etiological factors (1); the pathogenesis of CGCGs are not related to dental factors. In rare cases, the lesion may be localized near the teeth roots; thus it may be misdiagnosed as inflammatory odontogenic lesion especially if the associated tooth happened to be non-vital (7,9).

The review of similar literature revealed that CGCG localized to the periapical area most commonly occurred in the anterior mandible, in females, with an age of   years (6,7). According to the text book definitions, CGCG not localize to the periapical region, most commonly occurred in younger patients (10-30 years old) (10). Spatafore *et al*. researched 1659 periapical radiolucencies over a 10 year period and found that 52% of periapical lesions were granulomas, 42% cysts, 2% periapical scars and 4% other disorders (11). In 2005, De lange *et al*. reported that from 89 cases of CGCG, 79 cases were unilocular radiolucency and 8 cases (8.9%) were localized in periapical areas of the tooth (12).

Ortega *et al*. showed that from 43706 biopsy specimens 9.13% had endodontic pathosis of periradicular area, 26 cases had a histo-pathologic diagnosis of non-endodontic pathology. The most common periapical non-endodontic radiolucency was OKC (11 cases) followed by CGCG (3 cases). No malignancy was seen (13). Dehlkemper *et al*. described 16 cases of periapical CGCG. Lesions were most common in females,  30 year old and anterior segment of mandible. Most lesions had symptoms such as swelling and pain, recurrence was found only in one case after surgical treatment. Our case report concurs with this finding (6).

Nary *et al.* recently described a periapical CGCG in 16 year-old women, in lower incisor that was very similar to an inflammatory periapical radiolucency (7).

In this case study age, sex, and the location of the central giant cell granuloma agrees with the available literature, however the uncommon periapical location combined with the morbidity of the canine pulp made the diagnosis difficult. The usual diagnostic tests were not sufficient for accurate diagnosis and treatment. Lack of healing after 6 months caused surgical curettage to be carried out. Histopathologic examination revealed CGCG. As the origin of this lesion is not inflammatory, root canal therapy is not effective treatment for CGCG and will not resolve this lesion. Inclusion of CGCG in the differential diagnosis of periradicular radiolucency associated with non-vital tooth is necessary when periradicular lesions are refractory to endodontic treatment or the lesion recurs early in the same location. In these cases, histopathologic exam and periodic follow up is recommended.

A question then arises that should all teeth with pulpal pathosis and associated periapical lesion have routine surgical treatment including biopsy or should be conservatively treated with endodontic therapy but followed periodically? Though biopsy ensure definitive diagnosis, it is invasive and may have associated morbidity and complications such as bleeding, infection, and delayed healing (14,15).

## CONCLUSION

A differential diagnosis that includes CGCG’s should always be born in mind when assessing periradicular radiolucencies in the anterior mandible. Clinical examination, non surgical approach and periodic follow up is the recommended route for periradicular lesions. If endodontic therapy was ineffective at follow-up examination, surgical biopsy may be taken for histopathological examinations.
